# Clinical characteristics and outcome of hydrocephalus in neurosarcoidosis: a retrospective cohort study and review of the literature

**DOI:** 10.1007/s00415-021-10882-2

**Published:** 2021-11-07

**Authors:** Leroy ten Dam, Diederik van de Beek, Matthijs C. Brouwer

**Affiliations:** Department of Neurology, Amsterdam University Medical Center, University of Amsterdam, Amsterdam Neuroscience, Meibergdreef 9, 1105AZ Amsterdam, The Netherlands

**Keywords:** Neurosarcoidosis, Auto-immune disease, Hydrocephalus, Cerebrospinal fluid

## Abstract

Hydrocephalus is reported in approximately one-tenth of neurosarcoidosis patients. However, data on clinical characteristics and outcome are lacking. In this retrospective study, we present 11 patients with neurosarcoidosis and hydrocephalus on neuroimaging. Median age was 52 years and seven were female (64%). Presenting symptoms consisted of headache in 8 out of 11 (73%), vertigo in 5 (46%), gait abnormalities in 4 (36%), diplopia in 2 (18%) and decreased visual acuity in 1 (9%). Cranial imaging showed obstructive hydrocephalus in 10 (91%) and non-obstructive hydrocephalus in 1 (9%) out of 11, obstruction occurred at the level of the fourth ventricle in 6 out of 10 (60%). Treatment consisted of glucocorticoids in all the patients with additional methotrexate or azathioprine in 6 (55%) and infliximab in 1 (9%) patient. Neurosurgical intervention was performed in 10 out of 11 (91%) patients. Treatment led to remission, improvement or stabilization of disease in 9 out of 10 (90%) of patients. One patient died due to cerebral herniation despite neurosurgical decompression and CSF shunting. Median modified Rankin scale score at last follow-up was 2 (range 0–6). A systematic review and meta-analysis of studies on hydrocephalus due to neurosarcoidosis identified 36 patients that compared to our patients had a lower median age at onset and a higher mortality. Acute obstructive hydrocephalus due to neurosarcoidosis is a potentially fatal medical emergency requiring neurosurgical intervention and initiation of immunosuppressive therapy. If patients survive the initial phase, the outcome is generally favorable.

## Introduction

Sarcoidosis is an inflammatory disorder that can involve multiple organ systems [1]. Involvement of the nervous system is called neurosarcoidosis and has been reported in 5–20% of patients with sarcoidosis. Neurosarcoidosis can affect all parts of both the central and peripheral nervous system [2]. The diagnosis of neurosarcoidosis depends on a combination of clinical characteristics and results of ancillary investigations mostly used to rule out other disorders [3]. Hydrocephalus due to neurosarcoidosis was found to occur in 9% of patients with neurosarcoidosis in a meta-analysis [2, 4, 5]. There are no large series describing clinical characteristics of patients with hydrocephalus due to neurosarcoidosis and data regarding imaging characteristics, optimal treatment strategy and outcome are lacking. In this retrospective single-center cohort study, we evaluated clinical features and treatment response of patients with hydrocephalus due to neurosarcoidosis. We also performed a systematic review and meta-analysis of the literature regarding hydrocephalus in neurosarcoidosis.

## Methods

We retrospectively reviewed all case records from adult patients that were seen between June 2015 until June 2020 at the Neurology Department at the Amsterdam University Medical Centers a tertiary referral center for neurosarcoidosis. We included patients diagnosed with (1) neurosarcoidosis (either definite, probable or possible according to the criteria from the Neurosarcoidosis Consortium Consensus Group [3]) and (2) hydrocephalus on cranial imaging as reported by the neuroradiologist.

### Clinical data and ancillary investigations

Detailed history and neurological examination were retrieved from patient files in an electronic database including presenting symptoms, age of onset of symptoms, results of ancillary investigations and data on treatment and treatment response and outcome of hydrocephalus in neurosarcoidosis.

### Review of the literature

A literature search was performed in PubMed using the following terms: (“sarcoidosis” [MeSH Terms] OR sarcoid* [tiab] OR "Neurosarcoidosis" [Supplementary Concept] OR neurosarcoid* [tiab]) AND (“hydrocephalus” [MeSH Terms] OR hydrocephalus [tiab]). We included articles written in English describing one or more adult patients that were published in the last 25 years. The search was followed by a manual search in the reference lists of the publications found. The study conforms with the World Medical Association Declaration of Helsinki, 7th revision 2013 Fortaleza. Ethical approval is not required in the Netherlands for a retrospective study with anonymized patient data such as used in our study.

## Results

Between June 2015 and June 2020, 303 patients were analyzed because of suspected neurological involvement of sarcoidosis at our outpatient department or clinical department. Of these, 153 patients (50%) were diagnosed with neurosarcoidosis (either definite, probable or possible according to the criteria from the Neurosarcoidosis Consortium Consensus Group [3]). Hydrocephalus was present in 11 neurosarcoidosis patient (7%; Table 1) of whom 7 were female (64%). Median age at onset was 52 years (range 33–71 years).

**Table 1:** Clinical characteristics, results of ancillary investigations, treatment and outcome in hydrocephalus due to neurosarcoidosis

Characteristics	*n*/*N* (%)	Characteristics	*n*/*N* (%)
Median age at onset (range)	52 (33–71)	Biopsy	
Sex (female)	7/11 (64%)	Non-caseating granulomas	10/11 (91%)
History of sarcoidosis	5/11 (46%)	Classification of neurosarcoidosis [3]	
Median weeks to evaluation (IQR)	15 (3–38)	Definite	1/11 (9%)
Clinical symptoms at onset		Probable	9/11 (82%)
Headache	8/11 (73%)	Possible	1/11 (9%)
Vertigo	5/11 (46%)	Immunosuppressive treatment	11 (100%)
Gait abnormalities	4/11 (36%)	Corticosteroid pulse	7 (64%)
Diplopia	2/11 (18%)	Corticosteroid maintenance	9 (82%)
Decreased visual acuity	1/11 (9%)	Second-line therapy	4 (36%)
Abnormalities at first evaluation		Medication changes	5/11 (45%)
Impaired consciousness	4/11 (36%)	Corticosteroid therapy	3/5 (60%)
Papilledema	2/11 (18%)	Second-line therapy	5/5 (100%)
Nystagmus	4/11 (36%)	Third-line therapy	1/5 (20%)
Cranial nerve palsy	1/11 (9%)	Neurosurgical intervention	10/11 (91%)
Ataxia	2/11 (18%)	CSF shunt	8/10 (80%)
Sarcoidosis localisation		Ventriculostomy	3/10 (30%)
Lung	3/11 (27%)	Cyst fenestration	1/10 (10%)
Lymph nodes	4/11 (36%)	Follow-up	
Other	3/11 (27%)	Median duration in months (IQR)	23.5 (0–50)
Laboratory investigations		Median mRS score (range)	2 (0–6)
Elevated ACE	0/5 (0%)	Outcome of neurosarcoidosis	
Elevated sIL-2R	2/2 (100%)	Remission	2/11 (18%)
Elevated CSF leukocyte count	8/9 (89%)	Improvement	5/11 (45%)
Elevated CSF total protein	5/9 (56%)	Stable disease	3/11 (27%)
Cranial imaging		Death	1/11 (9%)
Communicating hydrocephalus	1/11 (9%)		
Obstructive hydrocephalus	10/11 (91%)		
Obstruction at 4th ventricle	6/10 (60%)		
Obstruction at foramen of Monro	3/10 (30%)		
Obstruction at cerebral aqueduct	1/10 (10%)		
Meningeal enhancement	7/9 (78%)		

The upper limit of normal of normal (ULN) of ACE is 40 U/L and the ULN of sIL-2R is 555 U/L. The ULN of cerebrospinal fluid analysis for leukocytes is 4 × 10^6^/L and for protein is 0.6 g/L

*ACE* angiotensin-converting enzyme, *CSF* cerebrospinal fluid, *IQR* interquartile range, *mRS* modified Rankin scale, *sIL2-R* soluble interleukin 2 receptor

Five patients (46%) had a history of sarcoidosis of which one had a history of neurosarcoidosis-associated myelitis (9%) and three used immunosuppressive therapy at symptom onset. Presenting symptoms were headache in eight (73%), vertigo in five (46%), abnormal gait in four (36%), diplopia in two (18%) and decreased visual acuity in one patient (9%). Time from symptom onset of hydrocephalus-related symptoms to first neurological evaluation was 15 weeks (interquartile range 3–38). Abnormalities on neurological examination included nystagmus and impaired consciousness each in four (36%), papilledema and ataxia each in two (18%) and bilateral sixth nerve palsy in one patient (9%). Neurological examination showed no abnormalities in two patients (18%). Median time from onset of symptoms to diagnosis of neurosarcoidosis was 4 months (range 0–34). Other organ systems involved were lymph nodes in four patients (36%), lungs in three (27%) and eyes, liver and bones, respectively, in one patient.

Serum angiotensin-converting enzyme (ACE) was analyzed in five patients and was elevated in none of them, soluble Interleukin 2 receptor (sIL-2R) was evaluated in two patients and was elevated in both. Cerebrospinal fluid (CSF) analysis was performed, commonly after external ventricular drain placement, in nine and showed an elevated leukocyte count in eight (89%) patients and elevated total protein in five (56%). One patient had a normal CSF analysis. CSF oligoclonal bands were present in the one patient in which they were tested.

**Fig. 1:**
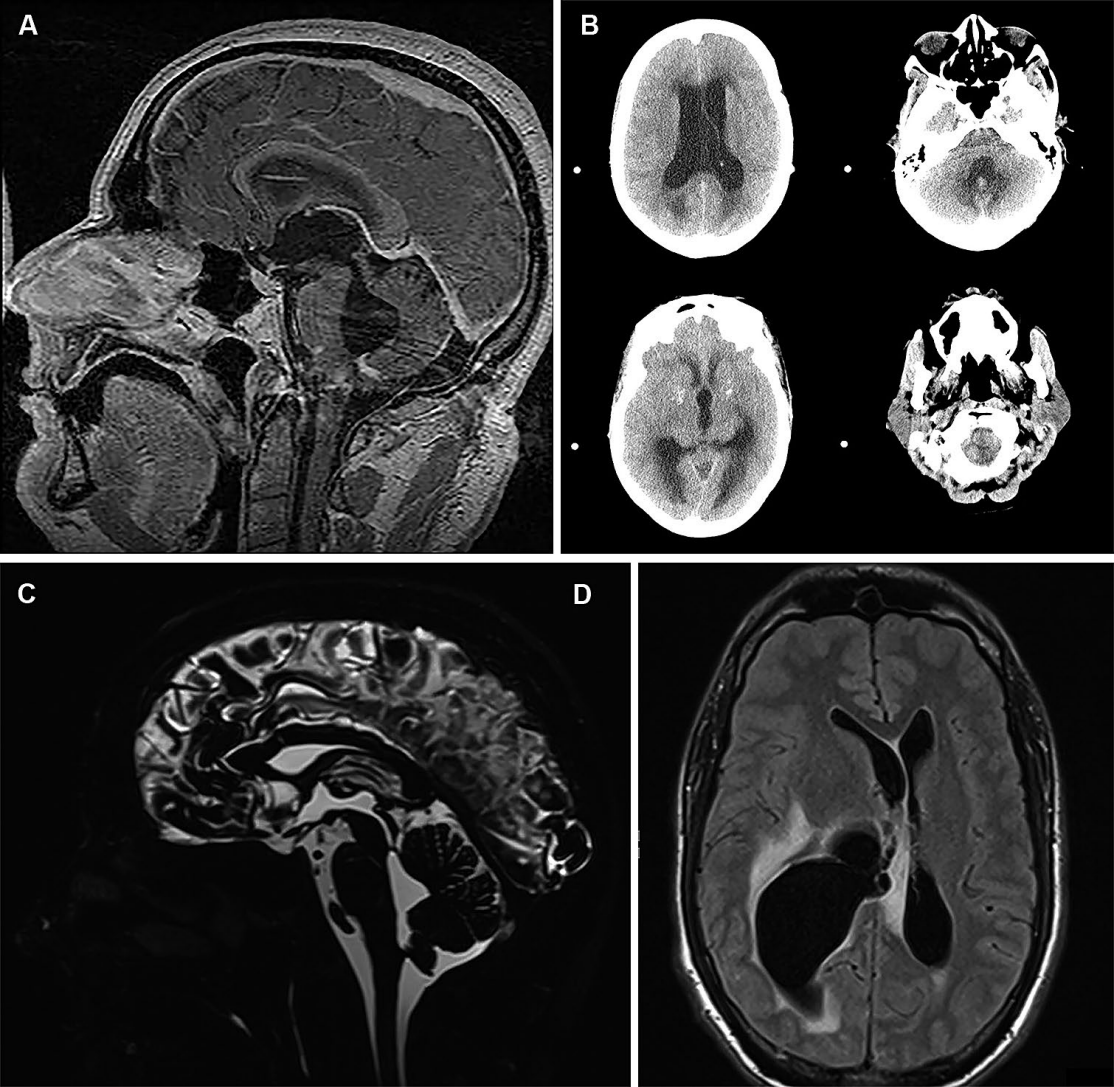
Obstructive hydrocephalus in neurosarcoidosis. Mid-sagittal contrast enhanced T1-weighted image (**A**) showing dilatation of the third and fourth ventricles and leptomeningeal enhancement of the bottom of the fourth ventricle. Axial CT scan (**B**) showing quadriventricular hydrocephalus and tonsillar herniation. Mid-sagittal T2-SPACE (**C**) showing a trapped fourth ventricle. Axial T2-FLAIR (**D**) showing dilatation of the right occipital horn due to a multicystic process located near the foramen of Monro

Results of cranial imaging were re-evaluated for all patients [ten magnetic resonance imaging (MRI) and one computed tomography (CT)] and showed hydrocephalus in all. The hydrocephalus was non-communicating/obstructive in ten patients and communicating in one. Obstruction was at the level of the fourth ventricle in six patients (60%), the foramen of Monro in three (30%) and the cerebral aqueduct in one (10%). In one patient, there was tonsillar herniation due to the obstruction hydrocephalus (Fig. 1). Post-contrast series were available in nine patients and showed abnormal leptomeningeal contrast enhancement in seven (78%). Abnormal contrast enhancement was located at the level of the fourth ventricle (foramina of Magendie and Luschka, surrounding the medulla oblongata or vermis of the cerebellum) in six patients (Fig. 1). Other abnormalities were a contrast enhancing multicystic process near the foramen of Monro, enhancing punctiform nodules in the brain parenchyma and dural contrast enhancement, which were all observed in one patient.

Fluor-18-deoxyglucose positron emission tomography (^18^FDG-PET) was performed in seven patients with no history of sarcoidosis, and showed abnormalities suggestive of sarcoidosis in all of them. Biopsy was performed in all patients. Leptomeningeal biopsy was performed in one patient and showed non-caseating granulomas (definite neurosarcoidosis), lymph node biopsy was performed in eight patients and showed non-caseating granulomas in seven (probable neurosarcoidosis) and was inconclusive in one (possible neurosarcoidosis), in one patient liver biopsy was performed and showed non-caseating granulomas (probable neurosarcoidosis). In the patient with possible neurosarcoidosis, chest CT was highly suggestive of sarcoidosis.

The median follow-up was 23.5 months (interquartile range 3–50 months). Immunosuppressive treatment was initiated in all 11 patients and consisted of corticosteroid pulse therapy in 7 (64%), corticosteroid maintenance therapy in 9 (82%) and second-line therapy, methotrexate or azathioprine, in 4 patients (36%). Neurosurgical intervention was performed in 10 out of 11 patients (91%) and consisted of external ventricular drain placement followed by ventriculoperitoneal shunt in 3 (30%), ventriculoperitoneal shunt in 5 (50%), ventriculostomy in 3 (30%) and cyst fenestration in 1 patient (10%). All but one patient were treated both with immunosuppressive agents as well as a neurosurgical intervention. Of these ten patients, three underwent neurosurgical intervention prior to immunosuppressive treatment, three received immunosuppressive treatment prior to neurosurgery and in four, neurosurgical and immunosuppressive treatments were initiated simultaneously. Medication was changed during follow-up due to insufficient response to initial therapy, side effects or a relapse of symptoms during follow-up in 5 out of 11 patients (45%). One patient had multiple relapses of symptoms. The relapse of symptoms of neurosarcoidosis was recurrence of hydrocephalus in four (80%) and meningitis without hydrocephalus and myelitis each in one patient (20%). Treatment changes included restarting or increasing the dosage of corticosteroid maintenance therapy in three (80%), corticosteroid pulse therapy in three (60%) and initiation of second-line therapy in five (80%; methotrexate in three and azathioprine in two). Third-line therapy (infliximab) was started in one patient (20%). Two patients underwent a second neurosurgical intervention because of failure of CSF shunt or ventriculostomy.

Neurological examination at the end of follow-up was available in ten patients and focal neurological deficits were present in only one patient and were attributable to sarcoidosis-associated myelitis. Outcome of neurosarcoidosis at last follow-up was classified as remission in two (18%), improvement in five (45%) and stable disease in three (27%). One patient (9%) died following cerebral herniation as a result of the hydrocephalus. Median modified Rankin scale (mRS) score at last follow-up was 2 (range 0–6).

### Literature review

Literature review yielded 95 articles of which 46 were assessed for eligibility based on title and abstract. Of these articles, five consisted of case series of neurosarcoidosis patients that included patients with hydrocephalus but did not report clinical characteristic, ancillary investigations, treatment and outcome of this subgroup [4, 6–9]. Seven articles were excluded based on language and one article reported on a patient that is included in our case series. Thirty-three articles describing 36 patients met our inclusion criteria (Table 2) [10–42]. The median age was 32 years (range 17–67) and 17 out of 35 patients were female (49%). Symptoms at onset were headache in 20 (56%), impaired consciousness in 16 (44%), nausea or vomiting in 14 (39%) and visual disturbances in 9 (25%) out of 36 patients. Elevated CSF leukocyte count was reported in 16 out of 22 (73%) and elevated total protein in 13 out of 21 (62%) patients. Cranial imaging showed hydrocephalus in all patients. The hydrocephalus was communicating in 9 (38%) and obstructive in 15 out of 24 (63%) of patients. Obstruction was at the level of the fourth ventricle in seven (47%), the cerebral aqueduct in five (33%) or above the level of the cerebral aqueduct in three patients (20%). Meningeal contrast enhancement was seen in 20 out of 25 patients (80%). Neurosarcoidosis was classified as definite in 20 (57%) and as probable in 15 (43%) out of 35 patients. Most patients were treated with steroids [31 out of 35 (89%)]. Second-line treatment consisted of azathioprine and methotrexate both in 4 of 35 (11%) patients. Third-line treatment with infliximab was started in two (6%) patients. Four out of 35 (11%) patients were started on other immunosuppressive agents which included mycophenolate mofetil, cyclophosphamide and cyclosporine. Neurosurgical intervention was performed in 29 out of 36 (81%) patients and included permanent CSF shunts in 21 (72%), ventriculostomy in 6 (21%) and other interventions such as endoscopic fenestration, partial lobectomy, open brain biopsy and temporary external CSF shunt in 9. Outcome of neurosarcoidosis was described as improvement in 27 (75%) patients, deterioration in 1 (3%) and deterioration leading to death in 8 out of 36 patients (22%). Four patients died due to cerebral herniation. In the other patients, cause of death was intractable seizures, treatment-related complications and pulmonary embolism.

**Table 2:** Clinical characteristics, ancillary investigations, treatment and outcome in patients with hydrocephalus due to neurosarcoidosis from the literature

Characteristics	*n*/*N* (%)	Characteristics	*n*/*N* (%)
Median age at onset (range)	32 (17–67)	Classification of neurosarcoidosis	
Sex (female)	17/35 (49%)	Definite	20/35 (57%)
Clinical symptoms at onset		Probable	15/35 (43%)
Headache	20/36 (56%)	Possible	0/35 (0%)
Visual disturbances	9/36 (25%)	Cumulative treatment	
Nausea or vomiting	14/36 (39%)	Corticosteroid therapy	31/35 (89%)
Impaired consciousness	16/36 (44%)	Methotrexate	4/35 (11%)
Laboratory investigations		Azathioprine	4/35 (1%)
Elevated CSF leukocyte count	16/22 (73%)	Infliximab	2/35 (6%)
Elevated CSF total protein	13/21 (62%)	Other immunosuppressants	4/35 (11%)
Cranial imaging		Neurosurgical intervention	29/36 (81%)
Communicating hydrocephalus	9/24 (38%)	CSF shunt	21/30 (70%)
Obstructive hydrocephalus	15/24 (63%)	Ventriculostomy	6/30 (20%)
Obstruction at 4th ventricle	7/15 (47%)	Other neurosurgical intervention	9/30 (30%)
Obstruction at cerebral aqueduct	5/15 (33%)	Relapse of symptoms	8/28 (29%)
Other site of obstruction	3/15 (20%)	Outcome of neurosarcoidosis	
Meningeal enhancement	20/25 (80%)	Improvement	27/36 (75%)
Enhancement at 4th ventricle	8/20 (40%)	Deterioration	1/36 (3%)
Enhancement at cerebral aqueduct	3/20 (15%)	Death	8/36 (22%)
Other site of contrast enhancement	15/20 (75%)		

*CSF* cerebrospinal fluid

## Discussion

Our study shows that hydrocephalus is a rare but potentially fatal manifestation of neurosarcoidosis that requires a combination of neurosurgical intervention and immunosuppressive treatment. In the absence of a positive history of (neuro)sarcoidosis, the differential diagnosis of acquired hydrocephalus is diverse and includes among others inflammatory, (post-) infectious, (post-)hemorrhagic and (post-)traumatic disorders and intracranial tumors such as colloid cysts or malignancy [5, 12, 43, 44]. To our knowledge, there is no literature regarding the percentage of hydrocephalus cases caused by neurosarcoidosis. As hydrocephalus was often the first manifestation of neurosarcoidosis ancillary investigations in acquired hydrocephalus with an unknown etiology should include sarcoidosis biomarkers (e.g., ACE and s-IL2R) and a chest CT and if normal ^18^FDG-PET. These investigations can help ascertain a potential biopsy site outside of the central nervous system. Histopathological evidence of non-caseating granulomas is necessary for a diagnosis of probable or definite neurosarcoidosis [3, 45].

Presenting symptoms of hydrocephalus in neurosarcoidosis are (sub)acute and related to increased intracranial pressure. Hydrocephalus in neurosarcoidosis is often non-communicating and caused by obstruction of the outflow from the fourth ventricle or of the cerebral aqueduct due to a chronic meningitis. Acute obstructive hydrocephalus can be fatal and an emergency neurosurgical intervention such as CSF shunting, ventriculostomy or decompression should be considered in all patients. Corticosteroid treatment in adjunct to neurosurgical intervention should be strongly considered in all patients. Intensification of immunosuppressive treatment is required in case of a relapse of symptoms, as a persistent or recurring granulomatous chronic meningitis can lead to an additional site of obstruction or shunt failure through obstruction of the CSF shunt [41]. Although relapses of symptoms and medication switches were frequent, there was stabilization, improvement or remission of neurosarcoidosis in nearly all patients that survived the initial phase.

As compared to our case series, the mortality was higher (9% versus 22%) and more patients had a diagnosis of definite neurosarcoidosis (10% versus 58%) in the review of the literature. The difference in mortality may be explained by publication bias as multiple articles reported on patients with sudden death. A previous review of the literature found an even higher mortality of 75% in patients with neurosarcoidosis who presented with seizures and had hydrocephalus [46]. However, mortality in this group was high (47%) irrespective of hydrocephalus. The higher percentage of patient with definite neurosarcoidosis was due to CNS biopsy being performed more often alongside neurosurgical treatment of the acute hydrocephalus in cases reported in the literature.

This study has several limitations. First, patients evaluated were referred to our tertiary center. This may introduce selection bias leading to overestimation of the impact of hydrocephalus in neurosarcoidosis. Second, the retrospective design of our study resulted in heterogeneous assessment of disease activity as well as missing data in some patients. This prohibits drawing firm conclusions regarding outcome and treatment effect. Third, as mentioned above there might be publication bias regarding hydrocephalus in neurosarcoidosis. Nevertheless, our retrospective single-center cohort study and systematic review provides valuable information on a rare disease as hydrocephalus in neurosarcoidosis.

In conclusion, hydrocephalus due to neurosarcoidosis is a medical emergency as onset is often (sub)acute and the hydrocephalus is obstructive in nature. Initiation of treatment should be prompt and include both neurosurgical interventions to lower intracranial pressure and immunosuppressive therapy to reduce granulomatous inflammation. If patients survive the initial phase outcome is generally favorable.

## Data Availability

The data that support the findings of this study are available from the corresponding author upon request.
